# A systematic review of RdRp of SARS-CoV-2 through artificial intelligence and machine learning utilizing structure-based drug design strategy

**DOI:** 10.55730/1300-0527.3355

**Published:** 2021-12-27

**Authors:** Fariha IMTIAZ, Mustafa Kamal PASHA

**Affiliations:** 1 Punjab University College of Pharmacy, University of the Punjab, Lahore, Pakistan; 2 Business R&D and Operations, Myst Enterprise, New Zealand

**Keywords:** RdRp, SARS-CoV-2, COVID-19, structure-based drug design, artificial intelligence, machine learning

## Abstract

Since the coronavirus disease has been declared a global pandemic, it had posed a challenge among researchers and raised common awareness and collaborative efforts towards finding the solution. Caused by severe acute respiratory coronavirus syndrome-2 (SARS-CoV-2), coronavirus drug design strategy needs to be optimized. It is understandable that cognizance of the pathobiology of COVID-19 can help scientists in the development and discovery of therapeutically effective antiviral drugs by elucidating the unknown viral pathways and structures. Considering the role of artificial intelligence and machine learning with its advancements in the field of science, it is rational to use these methods which can aid in the discovery of new potent candidates in silico. Our review utilizes similar methodologies and focuses on RNA-dependent RNA polymerase (RdRp), based on its importance as an essential element for virus replication and also a promising target for COVID-19 therapeutics. Artificial neural network technique was used to shortlist articles with the support of PRISMA, from different research platforms including Scopus, PubMed, PubChem, and Web of Science, through a combination of keywords. “English language”, from the year “2000” and “published articles in journals” were selected to carry out this research. We summarized that structural details of the RdRp reviewed in this analysis will have the potential to be taken into consideration when developing therapeutic solutions and if further multidisciplinary efforts are taken in this domain then potential clinical candidates for RdRp of SARS-CoV-2 could be successfully delivered for experimental validations.

## 1. Introduction

Scientists have actively put a considerable amount of effort to combat the global pandemic and have created an area of extreme interest to explore the novel antiviral drugs and vaccines. Understanding the structural proteins in the novel virus COVID-19 could encourage scientific community in their revelation of potent drugs by illuminating pathway involving disease [1,2]. The recent and most advanced method to accomplish this goal is the use of computational methods to unravel new entities in silico. In the last decennary from 2010 onwards, machine learning has offered cost-effective and fast implemented methods for the structural based drug discoveries [3,4]. If reasonable amount of related raw data is provided to a model using machine learning techniques, then patterns within the information can be identified, which eventually can help in identifying potential clinical candidates, through careful optimization of the further processing. If a specific protein is targeted, these models can predict inhibitor contenders using structural approach [5,6]. For COVID-19, the target genes selected from around the world are similar to those that contains RdRp [7,8]. In early diagnosis of SARS-CoV-2, RdRp as the target protein has evidently exhibited a remarkable performance as compared to other targets; therefore, further evaluation of RdRp has a substantial and discernible role in finding anti-COVID-19 drugs [9]. RdRp plays a pivotal role in RNA genome replication and is an imperative therapeutic target because the host does not have dependant equivalent to RdRp. Moreover, the target-related side effects are not expected due to lack of a counterpart to inhibited RdRp in host mammalian cells. Thus, RdRp is considered a striking target in the development and discovery of drugs [10]. For these reasons, this review focuses on the advances of RdRp protein of coronavirus entailing the structure-based drug design challenge using artificial intelligence and machine learning for the discovery of novel therapeutics against COVID-19.

## 2. Background

The first outbreak of virus from Coronaviridae family, causing pneumonia like symptoms, has been a global threat since 2002 [11]. The second outbreak was marked by SARS (severe acute respiratory disease) and MERS (Middle Eastern respiratory syndrome) in 2002 and 2013 respectively, causing pulmonary dysfunction and gastrointestinal problems [12]. While, SARS-CoV-2 caused the third outbreak that led to pandemic; creating more damage than ever before, the symptoms that varied from cold to respiratory failure and death [13]. This disease named COVID-19 has infected more than 20 million people worldwide, with death toll of about 3 million at the time of the review [14]. Presently, the virus is still spreading all over the world, and world’s population is hoping for particular anti-SARS-CoV-2 medicines along with vaccines [15,16]. Clinical scientists are considering an increasing number of drug prospects, and many trials have begun worldwide. So far, only remdesivir, a RdRp based medicine has been approved by FDA (Food and Drug Administration, United States of America) [17–19].

The structure of the coronaviruses reveals that it has envelopes with a single stranded RNA genome [20]. Further into this, the structure of RNA plays a crucial role in the life cycle of COVID-19. Amid other structural proteins, the intense modifications in viral spike have caused the high affinity of virus to the host receptor far more than SARS-CoV [21]. Coronaviruses have a complex of RdRp for the replication and transcription of their genes [22,23]. In RNA infections, genomic replication process is controlled by RNA dependant RNA polymerase, which virus inscribes [24]. After the cell is attacked by the virus, the genomic sequence of viral RNA is used as a template, and the protein synthesis for the translation of RdRp is done by host cell. As a result, RdRp completes the transcriptional process of various structural protein related mRNAs as well as viral genomic RNA. RdRp consequently can synthesize millions of nucleotides and hence facilitates virus to perform biological activities in the host cell [10]. This complex targets nucleoside analogue inhibitors. RdRp of coronavirus SARS-CoV-2 consists of catalytic subunit nsp12 with axillary subunits nsp7 and nsp8 respectively [22,25,26]. The structure of this RdRp is quite similar to the structure of RdRp of SARS-CoV that spread in 2002 [12,27]. In the single subunit of polymerase, the RdRp domain is similar to a right hand, comprising palm, thumb and fingers, where thumb represents subunits nsp7 and nsp8, and fingers as additional units of nsp8 [27–29]. Coronaviridae encode replication-transcription complex RTC into two open reading frames ORF1a and ORF1b, which are translated by genomic RNA. ORF1a encodes pp1a (polyprotein 1a) and both ORFs jointly encode pp1ab. The polyproteins 1a and 1ab are proteolytically processed into various nonstructural proteins called Nsps with the contribution of Mpro, an ORF1a encoded main protease, also named as 3CLpro. These Nsps joined into large RTCs that catalyzes replication and transcription of the genomic RNA. RTC core includes RdRp, which facilitates the whole process [30].

The RdRp protein varies from 240 kD to 450 kD and considered as conserved protein that could be a potential target for the development and discovery of antiviral drugs [31–34]. It can be safely concluded that targeting the RdRp site can lead to a therapeutically effective approach by restricting its region and inhibiting the viral replication [35].

Recent approaches using statistical tools such as molecular simulation and bioinformatics, by applying artificial neural network and machine learning techniques resulted in accelerated and stimulated production of active moieties and drugs that can be further investigated by clinical trials [13]. For example, structures, by implying machine learning using Q-UEL (XML-like Web Effort) can anticipate huge amount of real data to access pertinent and applicable literature. This technology can provide bioinformatics resources publically, available via internet, and a vast array of amino acids and proteins of multiple coronaviruses including SARS-CoV-2’s preserved sequences can be accessed in no time [2]. Q-UEL is a platform that aids in data mining in pharmaceutical and biomedical fields through artificial neural network by giving access to knowledge-based tags including probabilistic statements and general wisdom from encyclopedia, thesaurus and internet surfing [36]. Applying these methods may lead to the manufacturing of new synthetic anti-SARS-CoV-2 drugs and vaccines.

Bioinformatics with molecular simulation is playing indubitable role in finding treatment, diagnostic and preventive measures of COVID-19. The long haul journey in screening the active moieties have made simulation and discovery of new genetic sequencing and primers easy, economical and more precise with the help of artificial neural network.

The role of bioinformatics in combination with molecular simulation in the hunt for diagnostic, treatment and preventive methods of COVID-19 is unquestionable. Processes such as the screening of bioactive molecules, the simulation of biomacromolecule models, the discovery of primers and genetic sequencing compounds can be quicker, more precise and cheaper with the assistance of software based on artificial neural network. In view of the above, the present research aims to establish a systematic overview of structural data on all the efficient anti-SARS-CoV-2 agents based on RdRp of emerging RNA virus of SARS-CoV-2 using the approach of artificial neural networks and machine learning.

## 3. Data collection

**Figure 1:**
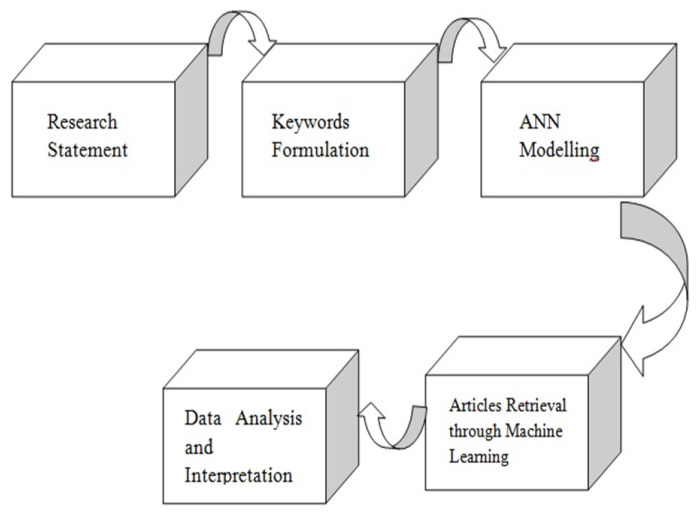
Framework of the research.

Data is collected through an extensive literature retrieval using artificial neural network technique from different research platforms including Scopus, PubMed, PubChem and Web of Science, through a combination of keywords with the support of PRISMA. “English language”, from year “2000” and “published articles in journals” were selected to carry out this research. The framework methodology adopted in this research is shown in Figure 1. Articles were screened using keywords “SARS-CoV-2”, “RNA dependant RNA polymerase”, “Artificial intelligence”, “structure based drug design” and “COVID-19” till July 2021. To make an arrangement between the articles based on these keywords ANN model was used which is explained below in detail (Figure 1).

**Figure 2:**
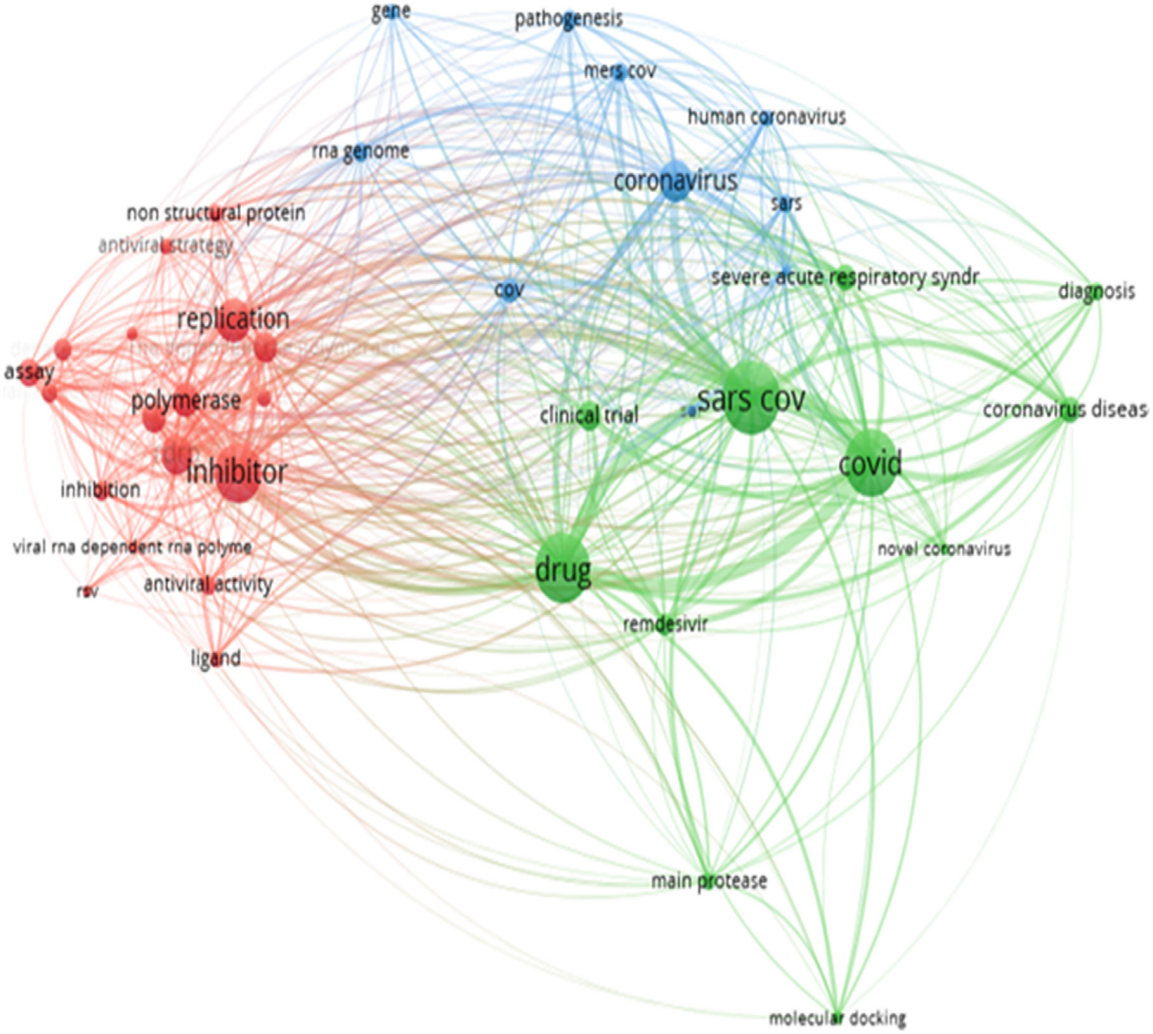
Keywords found in retrieved data related to RdRp sequences of coronaviruses RNA.

Endnote was used to extract database files and abstract screening was done using Excel software. Selected full text articles were screened for inclusion in the systematic review. The map was constructed using VOSviewer software entailing output information from ANN (Figure 2).

**Figure 3:**
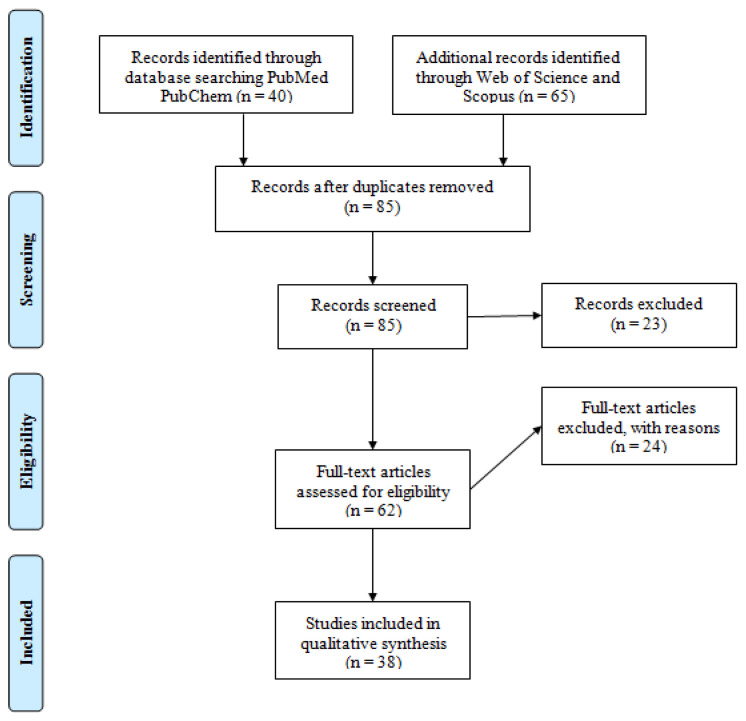
PRISMA flow diagram depicting criteria used for analysis of RdRp of SARS-COV-2 using structure-based drug design and AI.

After preliminary screening total of 105 articles were found, removing duplicates made them to 85. Abstract and title screening led to a total of 62 articles remaining. Full text screening excluded 24 articles and a total of 38 articles were included in this review. The PRISMA flow diagram is shown in Figure 3.

## 4. Result and discussion

The ANN data application has shown that while the number of RNA viruses that have triggered outbreaks in recent years is comparatively high. The emphasis in this study is on those viruses that we agree upon to have the highest therapeutic significance, social influence and scientific importance. The new coronavirus pandemic is disrupting global health services, and the research community is making an ongoing multidisciplinary attempt to resolve the immediate need for both care and prevention of serious acute coronavirus 2 (SARS-CoV-2) infections. SARS-CoV-2 case studies presented in this research may encourage the development of anti-RNA virus drugs. A detailed RNA based overview of coronaviruses is discussed below in detail.

### 4.1. Sequence of RNA based targeted coronaviruses

Data retrieved through machine learning emphasized that since RNA viruses are highly polymorphic so continuous surveillance is needed for new variants to be identified and distributed to new geographical areas or populations of patients. Therefore, it is also a challenge to update the awareness of RNA viruses such as SARS-CoV, SARS-CoV-2, and MERS-CoV. Furthermore, veterinary virology is an important part of this activity, in order to promote the ability to characterize zoonotic viruses, breaching the species boundary and entering the human population. When a novel human pathogen is suspected or confirmed, it is a significant step towards the control of the disease and outbreak to acquire partial or full genome information.

Data collected and analyzed utilizing ANN confirmed more than 14,000 nucleotide sequences of SARS-CoV-2 in NCBI GenBank, mostly collected from cities in USA and China. The full sequence of the gene of SARS-CoV-2 virus was first published by scientists from Adolf Lutz Institute and University of Sao Paulo in collaboration with Oxford University in February 2020 in GenBank accession number MT126808 [37]. One of the first cases of respiratory infection in China, caused by a new virus led to the series of experiments that established the identity of this new virus by analyzing its RNA sequences collected from the fluid samples of lungs, that connected it from Coronaviridae RNA family. The genetic sequencing technique is helpful in understanding the genome of a population, and can be declared as a starting point to understand the role and structure of its genes. ANN acts as a house of genome of microorganisms such as SARS-CoV-2, it not only gathers data from a specific region, but also collects the sequencing from patients from all over the world, and it also had made it possible to track the profile of infectious disease, its spread in different nations, and eventually helping in finding disease-fighting techniques and mapping the mutation rate [13].

Another method for the determination of the key structural models of SARS-CoV-2’s RNA translated polyproteins is by acquisition of crystallographic particulars using x-ray diffraction technique [38]. These structural models were then assessed by already available structural model of SARS-CoV in Protein Data Bank (PDB), which calculated 96% sequential identification. Furthermore, the structural data of SARS-CoV-2’s RNA protein, obtained by cryogenic electron microscopy, helped in the development of models by using SARS-CoV glycoproteins as a reference [39]. The structural details obtained by these methods can act as a guide to understand the actual configuration under the predefined experimental conditions. The study of the viral RNA structure with the assistance of computational methods and machine leaning, the main sockets of interaction between the enzyme and the inhibitor were possible to visualize at molecular level. This could be related to the direct improvements and amendments in the structures of RNA, and inhibitory function of the drugs can be easily modified [38].

The urgency of successful anti-COVID-19 therapy led to the virtual screening methods of selecting bioactive moieties by using artificial intelligence. This ended up improving this road of discovery, by promoting the initial selection of molecules with similar steric and electronic properties, which is also known as ligand-based drug planning. On the basis of these, it has been made possible to pick potential inhibitory targets, agonists and antagonists from virtual databases of compounds against a single strand of RNA this will lead to impact compounds with targeted structural changes [39].

In the lifecycle of coronaviruses, conserved structured elements play important functional roles [40]. These structural elements add complexity to the regulatory functions that encode viral RNA by directly interacting with helicases and RNA-binding proteins. If the functions of these structured elements are disrupted, this can lead to unexplored strategy in which decreased viral loads will have minimum to no effect on normal biological cells [41]. Despite the existence of this idea around 6 years ago, the advances in computational modeling and artificial intelligence has aided it in overcoming the critical barriers by a quantum leap in high throughput RNA structure analysis [42].

Many functionally validated viral families, including coronaviruses, have identified highly conserved RNA structured elements such as 5’UTRs and 3UTRs that highly impact the viral replication [40, 43–45]. A total of 106 regions in these RNA elements have already been reported that can be a potential target for novel antiviral drugs [46].

### 4.2. RdRp and SARS-CoV-2 in light of ANN

Digital libraries of compounds such as ZINC provide a huge variety of structures and sources, which helps in finding new lead compounds via high throughput screening, which ultimately increases the quality of the work and eases search process. The databases can be explored to classify potential anti-RdRp molecules. Researchers selected more than 300 compound from these digital libraries, which were then docked against proteases (PDB ID 6LU7), and their interaction energy of target-ligand bond was evaluated [47].

The evolution in virtual screening has led to the addition of three-dimensional bimacromolecular structural models in the databases which are now publically available to analyze data using molecular simulation tools. PDB now has more than 160,000 three-dimensional structures in its library (Table).

**Figure 4:**
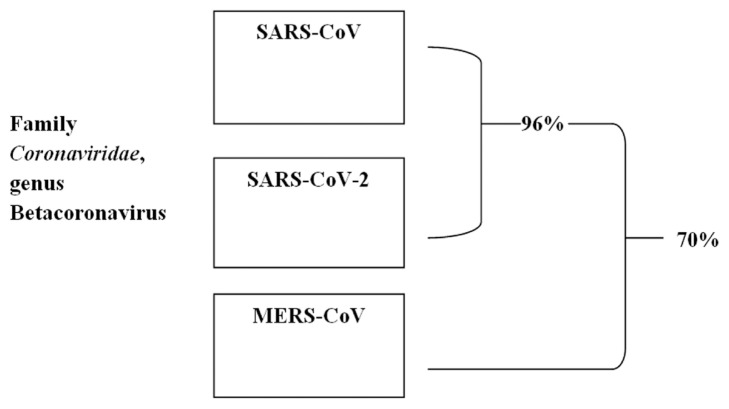
Schematic representation of the RdRp identity percentages shared by RNA viruses of the same and different clusters.

SARS-CoV-2, discovered by a metagenomic method, sequenced and identified as a new member of the Coronaviridae family based on sequence homology, is a notable example of this kind [48]. Undoubtedly, the discovery of new mutations and varieties of viruses has been encouraged by the rapid progress of whole-genome sequencing by introducing a variety of next-generation sequencing methods, i.e. machine learning and artificial intelligence, allowing full-length sequencing at a fraction of the time for the discovery [49–52]. The genetic study of the RNA viruses considered in this review is computed in a formal description of the RdRp sequence of the various species. RdRp sequences can then be split into three clusters, which are especially linked to the family and genus to which they belong (Figure 4).

SARS-CoV-2’s RdRp shares the highest amino acid identity (96 percent) with SARS-CoV’s RdRp among coronaviruses, while MERS-CoV’s RdRp homology is only 70 percent. While the main method for monitoring outbreaks and virus evolution is whole-genome sequencing, genome processing is just the first step in developing and evaluating antiviral drugs. Indeed, to determine the drug capacity of a possible target protein, structural specifics are important and may be provided by structural biology attempts or, to a less precise but more immediate extent, by homology modeling [53,54].

Using a machine learning method, we analyzed that ClustalX version 2.1 with the pair wise alignment algorithm, the sequence alignment retained the default amino acid color scheme, i.e. blue = hydrophobic; red = positive charge; magenta = negative charge; green = polar; pink = cysteine; orange = glycine; yellow = proline; cyan = aromatic; white unconserved or gaps [34]. In this study, the black bar below the alignment corresponds to the degree of conservation: at any position, the higher the bar, the higher and the conservation. We conclude that the amino acids that are retained in the aligned sequences are indicated. In particular, this contributed to a deeper understanding of the structural characteristics of the emerging coronavirus RdRp, which is discussed below.

### 4.3. Structure of coronaviruses RdRp in silico

**Figure 5:**
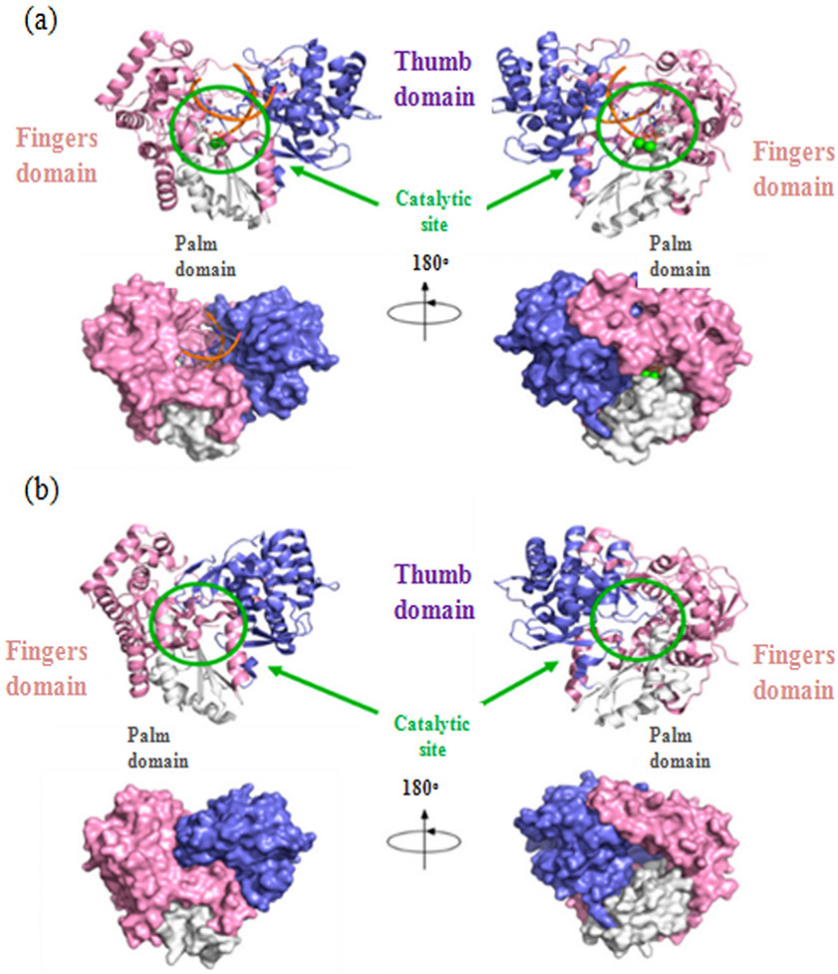
Cartoon representation of thumb (blue), palm (white) and fingers (pink) subdomains (a) RNA-dependant RNA-polymerase, (b) apo RdRp (Source: [54]).

The ultimate shape of SARS-CoV-2 RdRp resembles a closed right hand with the sub domains of the palm, thumb, and finger, much like all other polymerases. The fingers are further classified into index finger, middle finger, ring finger and pinky finger [55]. The palm area conserves catalytic site while fingers and thumb are subdomains that makes two tunnels and meet at the catalytic site. It has a nidovirus-specific domain on the N-terminal tail with nucleotidyltransferase activity [53]. Unfortunately, only a few SARS-CoV RdRp crystallographic structures have been solved to date, while no MERS-CoV RdRp structures are available. In comparison, Gao et al. (2020) recently provided detailed details on the apo RdRp structure as well as the elucidation of the conformational modifications of the protein by binding to RNA and a nucleoside analogue inhibitor. Within less than a year of the SARS-CoV-2 outbreak, the Cryo-EM technique solved nine three-dimensional structures of its RdRp (Figure 5) [44].

### 4.4. Drugs against RdRp-ANN approach

In the ongoing pandemic, scientists from all over the world proposed the use of already marketed antiviral drugs against COVID-19. The efficacy of these drugs turned out to be limited. Other reports showed that the use of preexisting antiviral drugs, suggested by health service providers, is a cost effective and time saving initiative as the de novo drug discovery takes years while the mortality rate is increasing day by day [35]. The following few drugs have tested against COVID-19 in recent times including remdesivir, ribavirin, corticosteroids, lopinavir-ritonavir and interferons [21,56].

**Figure 6:**
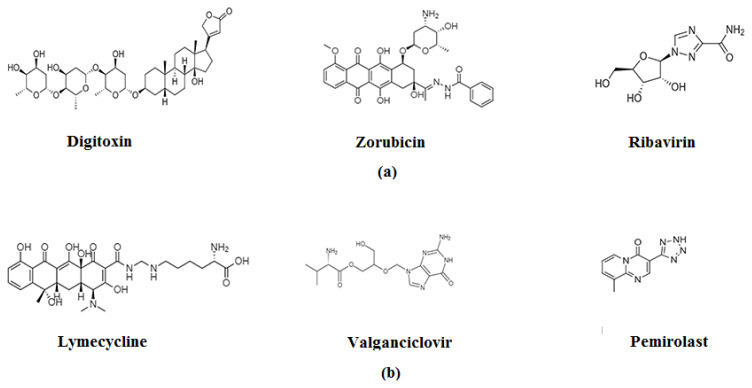
Molecular agents obtained from virtual libraries with anti-SARS-CoV action (Source: [2]).

Using ANN and molecular modeling, the anti SARS-CoV-2 agent PubChem CID 444745 compound (Figure 6), has demonstrated the enzyme inhibition potential with protease forming the most stable complex. Digitoxin is a cardiac glycoside; however, currently it has been delineated as antiviral agent that is active against coronavirus [57]. Structure of digitoxin explains it has a steroidal nucleus which shows involvement of glycosidic linkage through oxygen atom. Glycoside’s aglycone type not only reveals its physicochemical characteristics but also shows various therapeutic uses [58]. Numerous studies have shown digitoxin’s anti-COVID-19 potential using structure-based screening of different databases [59–61].

The ZINC databases of drugs were virtually examined for their interaction with various possible molecular targets of the virus, emphasizing the promise of zorubicin against SARS-CoV-2’s glycoprotein, ribavirin against PLpro; lymecycline against 3CLpro; and valganciclovir against RNA-dependent RNA polymerase (Figure 6) [62]. Studies have revealed that spike proteins are new hotspot for viral mutations, and they help in either transmission or in enhanced binding by altering the receptor-binding domain RBD. Zorubicin have virtually shown promising anti-COVID-19 results by specifically binding to RBD and inhibiting the binding of S-protein [63]. Likewise, the docking model of PLpro with ribavirin showed the inhibition of PLpro by forming hydrogen bond between Gln270, Gly164, Tyr274 and Asp303 [64]. Only the possible inhibitor of drugs or substances in clinical trials with anti-HCV activity has been tested in silico against SARS-CoV-2 RdRp is ribavirin. On the other hand, lymecycline has shown remarkable affinity to 3CLpro. 3CLpro is also known as Nsp5, which produces mature enzymes to cleave downstream Nsps on 11 sites which releases Nsp14-Nsp16. 3CLpro conciliates maturation of Nsps, which is vital for virus life cycle, hence lymecycline is an attractive target in the development of SARS-CoV-2 drugs [62]. Valganciclovir has also exhibited good binding affinity with PLpro as well as RdRp [62,65]. While docking studied on pemirolast revealed that they have tremendous binding affinity with S-protein as well as ACE2 inhibitors [66].

National Medical Products Administration of China issued the approval of use of favipiravir and reported it the first antiviral drug against SARS-CoV-2 [67]. A number of other drugs such as ribavirin, remdesivir, sofosbuvir, and galidesivir are under clinical trials for their anti-RdRp activity [68–75].

### 4.5. RdRp and remdesivir

Remdesivir has been using against Ebola virus and it has clinically been proven that it targets RdRp by inhibiting the viral RNA synthesis [74,76,77]. In a recent study, it has been concluded that remdesivir can bind to RNA-binding channels of SARS-CoV-2 [62]. Recent research on structural studies of RdRp has revealed promising use of remdesivir by inhibiting the nucleotide analogue and provided a structural template to further investigate potential antiviral drugs against COVID-19 [18,78].

### 4.6. Anti-RdRp drugs using molecular docking

Ahmad et al. [79] screened all the marketed drugs and the drugs that are in clinical trial and reported that all the FDA approved drugs have stable interaction and lowest binding energies with the key residues. This concluded that these drugs have high potential of inhibiting the activity of RdRp. The most promising out of these drugs that interacted with single core RdRp are Argiprestocin, ornipressin, carbetocin, otosiban, demoxytocin, lypressin, examorelin and polymyxin B1. While the compounds that interacted with the complex of RdRp are cistinexine, pegamotecan, nacartocin, cisatracurium, ebiratide, diagastrin and benzquercin. Among these, the top candidates that showed strong structural binding efficacy with both single core and complex RdRp are lypressin, polymyxin B1 and ornipressin.

Another report predicted that small molecule inhibitors can impressively target coronavirus’s RdRp by using molecular docking technique. The study reported that the guanosine triphosphate GTP site has the close proximity to RNA primer and RNA template as well as in the active region between palm and thumb. As the RdRp functions both primer independently and dependently, thus, depicts the dual functionality of GTP sites [80]. The initiation of primer independent RNA replication process, this site incorporated GTP as second nucleotide [81]. While in case of primer dependant initiation process, prior to the summation to the RNA chain, this site can be processed as a potential nucleotide binding site for an upcoming nucleotide. These two modes of primer assisted and unassisted RNA replication chains can be impeded by use of small molecules, hence, making RdRp dysfunctional [80].

## 5. Conclusion

Based on the evidence provided in this study, it is highly plausible that if multidisciplinary and concentrated efforts are committed to this task, drugs operating on the RdRp of coronaviruses could be successfully produced. The in silico research for small molecules is also intended to boost the resilience of health services and foreign organizations for potential future pandemics. In this respect, the lack of structural information on catalytic-competent or ligand-bound RdRps distinguishes SARS-CoV-2 and could hinder the effective use of structure-based drug design in both repositioning and traditional approaches. Overall, in this review, we summarized the recent findings in targeting the RdRp of RNA viruses through machine learning and found the existing studies fail to resolve a unified approach. In this regard, our study successfully produced an aggregated response in the past to aid the process further. We found that there is no general agreement among the researchers in this domain. Together with structural hints, the aggregated literature discussed here should motivate the design of additional small molecules and set the foundation for advanced structure-based approaches. However, to improve the strategies and methodologies for novel research, there is still a need of sophisticated AI tools and data visualization for not only decision making but also for future global outbreaks.

## Figures and Tables

**Table:** AI tools used to access different hence sequences for SARS-CoV-2.

No	AI tool	Number of gene sequences found for SARS-CoV-2	Best suitable antiviral drug	Gene sequence	Reference
1.	NCBI GenBank	14,000 nucleotide sequences	-	GenBank accession number MT126808	[37]
2.	Crystallographic data via X-ray diffraction	96 percent sequential identification	Viral RNA translated polyproteins complexed with the alpha-ketoamide 13b inhibitor	SARS-CoV spike glycoprotein	[38]
3.	Virological.org and GISAID	300 compounds studied,	-	PubChem CID 444, 745 compound	[47, 82]
4.	ZINC	-	Ribavirin against PLpro; lymecycline against 3CLpro; and valganciclovir against RNA-dependent RNA polymerase (RdRp)	-	[47]
5.	The Protein Data Bank (PDB-www.rcsb.org),	160,000 structures (three-dimensional biomacromolecular structure models)	-	(PDB ID 6LU7)	[39, 83]
